# Solid Variant of an Aneurysmal Bone Cyst of the Thoracic Spine

**DOI:** 10.7759/cureus.1208

**Published:** 2017-05-01

**Authors:** Varshil Mehta, Pravin Padalkar, Maya Kale, Ambadas Kathare

**Affiliations:** 1 Department of Internal Medicine, MGM Medical College, Navi Mumbai, India; 2 Department of Orthopedics and Spine, Center for Orthopaedic & Spine Surgery, New Panvel, India; 3 Department of Microbiology, MGM Medical College, Navi Mumbai, India

**Keywords:** abc, osteolytic lesion, bone tumor

## Abstract

The solid variant of an aneurysmal bone cyst (ABC) has been observed very rarely, especially those involving the spine. In this case report, we present a very unusual tumour of the thoracic spine which was managed by 360˚ decompression via posterior-only approach and stabilization.

A 16-year-old boy presented to us with a sudden onset of weakness in both lower limbs leading to paraplegia. He also had a history of back and chest pain over the past one year. A collapse of the T5 vertebrae on plain radiograph was observed. The patient was immediately shifted to the operation theatre with an initial plan of a total en bloc spondylectomy of the T5. However, intraoperatively, histology favored a solid-ABC variant rather than a spindle cell tumour or giant cell tumour. Thus, the initial plan was revised to a 360˚ decompression without resecting the body en bloc via a posterolateral approach.

After surgery, complete resolution of his sensory and motor dysfunction was achieved. His chest and back pain also resolved. The diseased vertebral body gradually healed and new bone formation was seen at 18 months postoperatively.

This case report concludes that a solid variant of an ABC should be considered as a differential diagnosis for tumours involving the spine. An intraoperative frozen section procedure should be undertaken, especially during emergency situations. Early diagnosis and appropriate surgical management play an important role in the successful management of a solid variant of ABC.

## Introduction

The solid variant of an aneurysmal bone cyst (ABC) is a very rare case which generally accounts for about 3.4% to 7.5% of all ABCs [1]. It is a cystic lesion that can occur and expand in any part of the bone, often affecting individuals in the second decade of their lives [2]. A definitive diagnosis is impossible but there are radiographic characteristics that are highly suggestive of ABCs on computed tomography (CT) and magnetic resonance imaging (MRI) scans.

Jaffe and Lichtenstein first described ABC in 1942, when they noted, "a peculiar blood-containing cyst of large size" [3]. As many as 69% of primary ABCs demonstrate a characteristic clonal t(16;17) genetic translocation which can lead to an upregulation of the TRE17/USP6 oncogene [4].

Although benign, an ABC has the potential to grow acutely. Its expansile nature can cause a lot of swelling and pain while disrupting the growth plates. The changes in the bone can also lead to deformity and pathological fractures. It can also cause some neurological manifestations depending upon its location [2]. A distinct solid variant of ABC was first reported by Sanerkin, et al. in 1983 [5]. This type of solid variant could be easily misdiagnosed as a spindle cell tumour, especially osteosarcoma [1].

Symptomatic ABCs are generally treated with surgery. However, asymptomatic ABCs (characterized by clinically insignificant destruction of the bone) are generally left alone with just close monitoring for any abnormal changes [6].

## Case presentation

**Figure 1:**
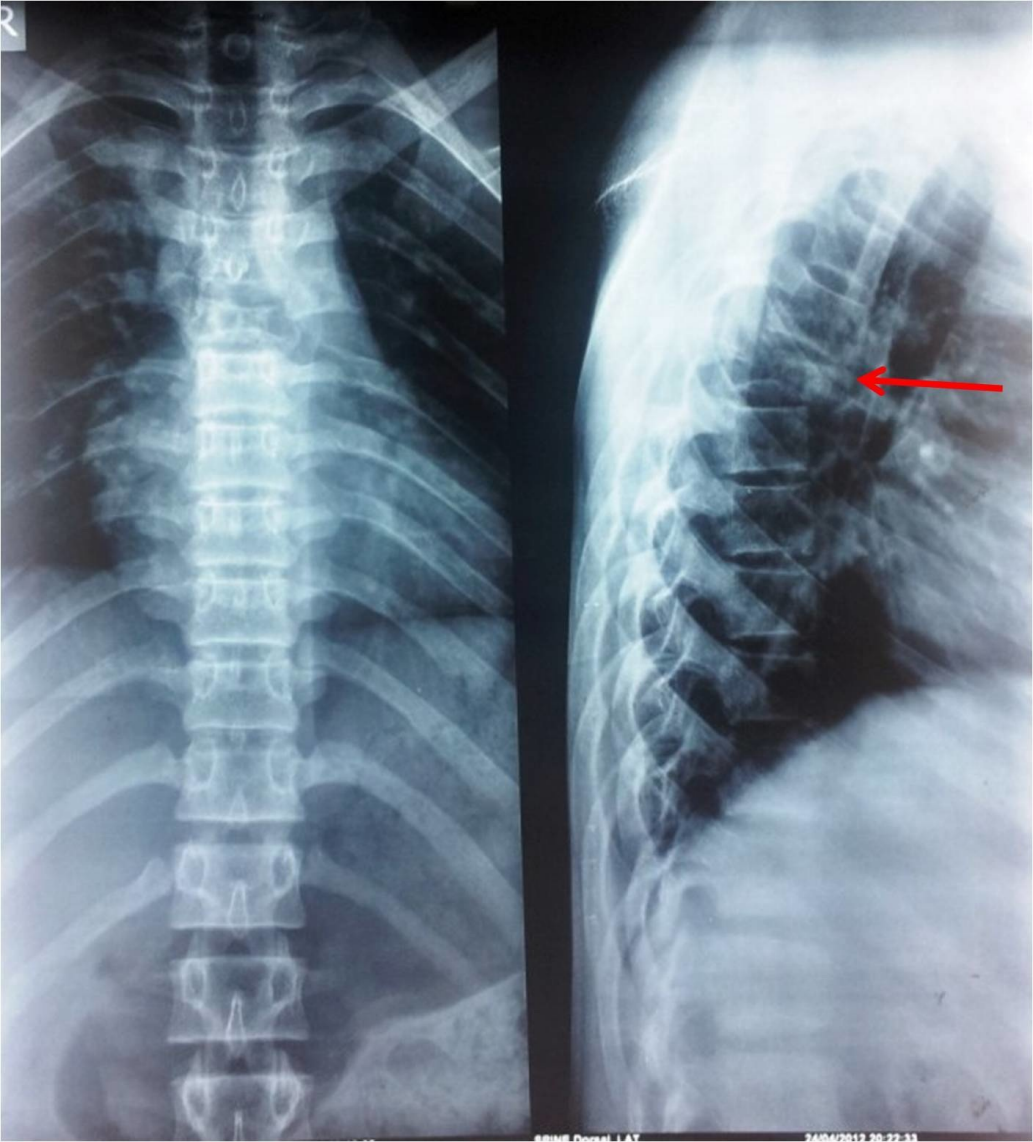
Radiograph showing collapse of T5 vertebra Anteroposterior and lateral view x-ray of the thoracic spine showing pathological fracture and collapse of T5.

A 16-year-old boy was presented to us with a sudden onset of weakness in both lower limbs leading to paraplegia. He also had a history of back and chest pain over the last one year. A good rectal tone without any perineal anesthesia was noted in the rectal examination and the post-void residual urine volume was negligible. All laboratory findings were within normal range. Plain x-ray showed the collapse of the T5 vertebra (Figure 1).

**Figure 2:**
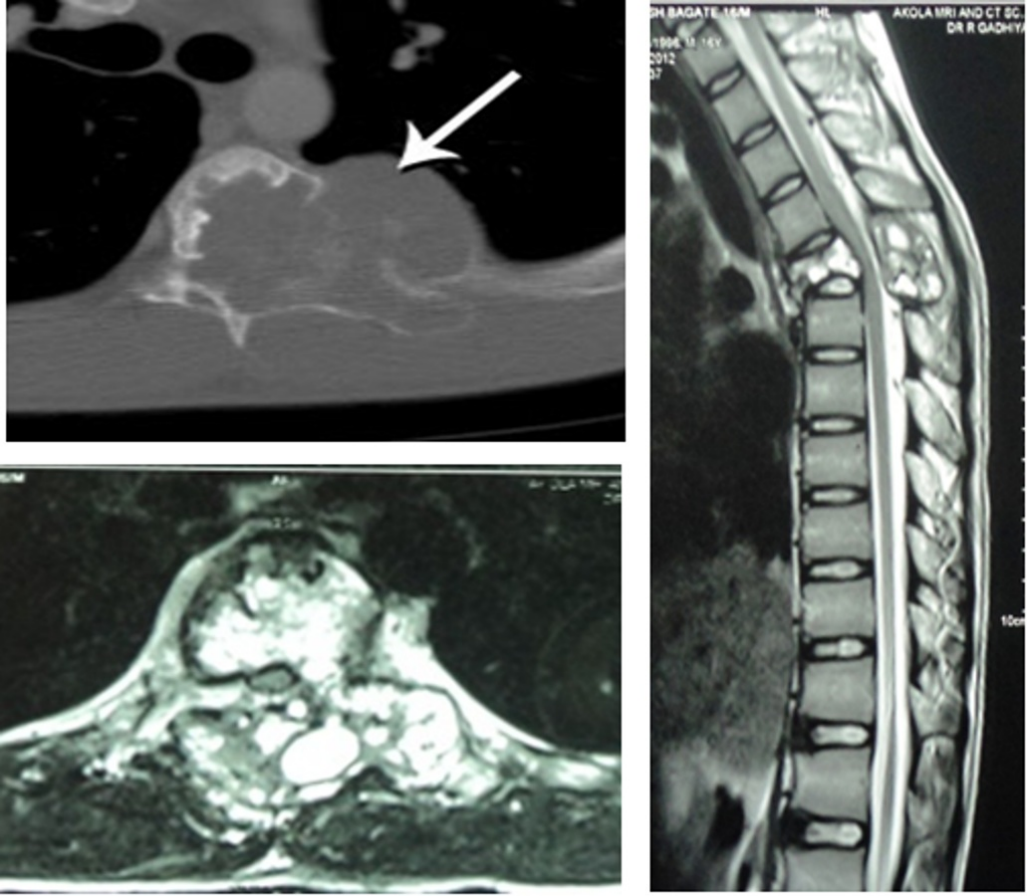
CT (top left) and MRI (bottom left and right) Computed tomography (CT), magnetic resonance imaging (MRI).

CT axial images showed an expansile and lytic lesion in the vertebral body, left pedicle, and transverse process. T2-weighted sagittal and axial MRI T2-weighted images of the thoracic spine showed hyperintense signals in the T5 vertebral body, left pedicle, and transverse process with a pathological fracture (Figure 2).

A preoperative biopsy was not successful because of the urgent requirement of decompression. The patient was taken to the operating room on an emergency basis with an initial plan of a total en bloc spondylectomy of the T5.

**Figure 3:**
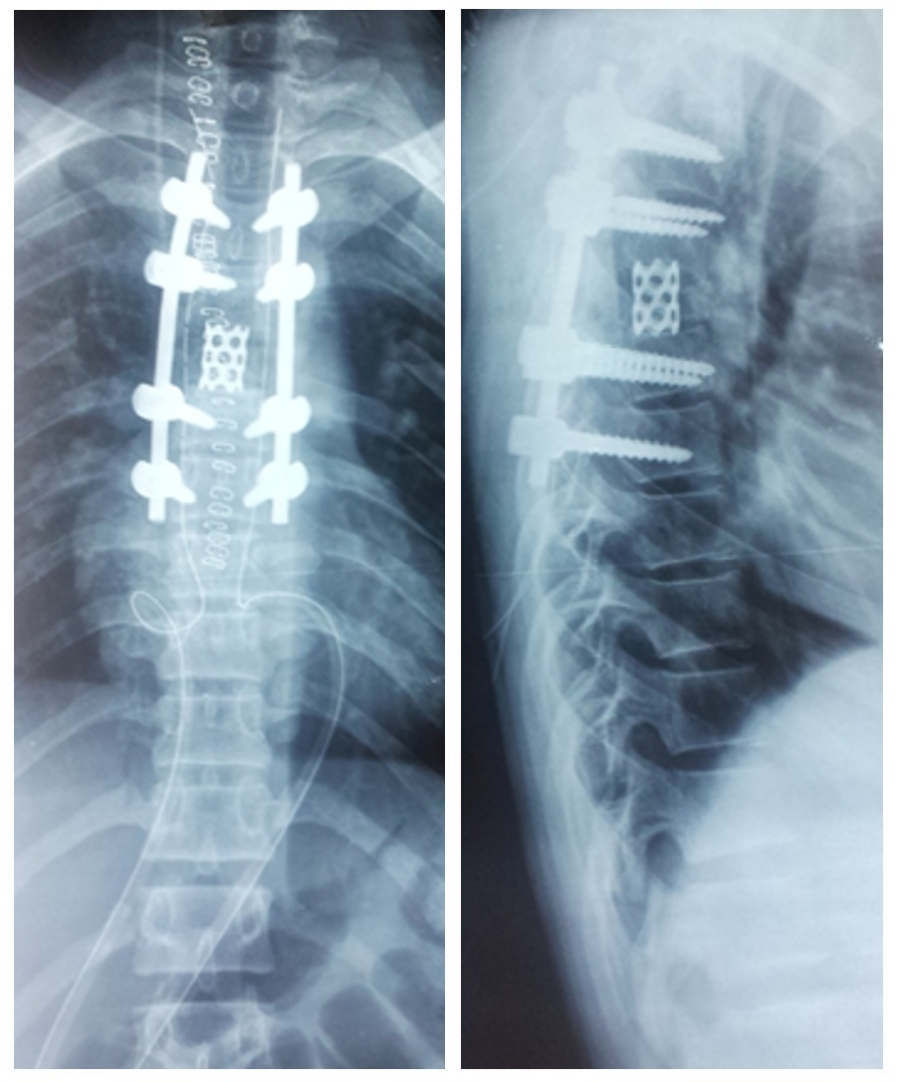
Intraoperative anteroposterior and lateral view x-ray of the thoracic spine showing cage and pedicle screw fixation

Intraoperatively, histology favored a solid-ABC variant rather than a giant cell tumour. Thus, the initial plan was revised to palliative surgery with a 360˚ decompression (without resecting the body) en bloc via a posterolateral approach as shown in Figure 3. The vertebral column was reconstructed with an expandable titanium cage (inserted via a costotransversectomy approach) and pedicle screw fixation in a 360˚ manner (Figure 3).

### Results

**Figure 4:**
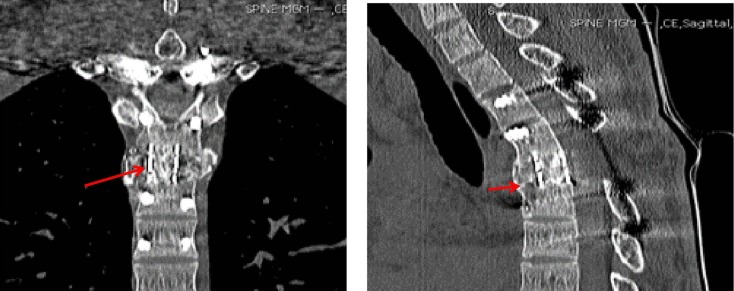
Postoperative MRI showing new bone formation at 18 months Magnetic resonance imaging (MRI).

After surgery, a complete resolution of sensory and motor functions was achieved. The pain in his chest and back also resolved gradually within a few days. Also, the diseased vertebral body gradually healed and new bone formation was seen at 18 months postoperatively (Figure 4).

## Discussion

ABCs, although benign, are locally very aggressive and highly vascularised tumours. Generally, post-surgery, it takes a long time to obtain a healing state and new bone formation with a recurrence rate of about 50% [7]. However, Malghem, et al. concluded that spontaneous healing is possible [8].

ABCs are found to have a predilection for the lumbar spine, which was reported by Boriani, et al. [9]. Although CT and MRI scans are the preferred diagnostic methods, a biopsy is the utmost necessary prerequisite for confirming the diagnosis, due to its similarity in appearance with many other bone lesions. The biopsy generally shows proliferating round or oval cells, generally mixed with multinucleated giant cells distributed randomly. It also contains regions of reactive fibroblastic proliferation and microcystic components filled with blood [10].

Depending on the proliferative component, the solid variant of an ABC is often misdiagnosed histologically for other benign or malignant tumour-like bone lesions [1]. The pathological differential diagnosis (solitary bone cyst, giant cell tumour, hemangioma, osteosarcoma, and chondroblastoma) should always be kept in mind while thinking of ABC [10].

The treatment of ABC is also very controversial which includes arterial embolization, curettage with or without bone grafting, complete excision, intralesional drug injections (steroid and calcitonin), and radiation. However, early diagnosis and appropriate surgery play the most important role in the successful management of ABC [10].

Whether surgical management results in a better outcome and lower recurrence rate than other methods (conservative or palliative; for example, curettage alone) remains controversial. Hence, more studies comparing these methods should be conducted.

## Conclusions

This case report concludes that an ABC should be kept as a differential diagnosis for tumours involving the spine. Intraoperative frozen sections should always be performed when there is a doubt in mind and in cases of emergencies. An effective spinal decompression and stabilization of the ABC can be achieved by partial or subtotal excision. However, for the successful management of ABC, early diagnosis and appropriate surgical management should be considered.

## References

[1] Bertoni F, Bacchini P, Capanna R, Ruggieri P, Biagini R, Ferruzzi A, Bettelli G, Picci P, Campanacci M (1993). Solid variant of aneurysmal bone cyst. Cancer.

[2] Clayer M (2008). Injectable form of calcium sulphate as treatment of aneurysmal bone cysts. ANZ J Surg.

[3] Jaffe HL, Lichtenstein L (1942). Solitary unicameral bone cyst: with emphasis on the roentgen picture, the pathologic appearance and the pathogenesis. Arch Surg.

[4] Panoutsakopoulos G, Pandis N, Kyriazoglou I, Gustafson P, Mertens F, Mandahl N (1999). Recurrent t(16;17)(q22;p13) in aneurysmal bone cysts. Genes Chromosomes Cancer.

[5] Sanerkin NG, Mott MG, Roylance J (1983). An unusual intraosseous lesion with fibroblastic, osteoclastic, osteoblastic, aneurysmal and fibromyxoid elements. "Solid" variant of aneurysmal bone cyst. Cancer.

[6] Tedesco Tedesco (2017). Aneurysmal Bone Cyst. http://emedicine.medscape.com/article/1254784-overview#a11.

[7] Ruiter DJ, van Rijssel TG, van der Velde EA (1977). Aneurysmal bone cysts. A clinicopathological study of 105 cases. Cancer.

[8] Malghem J, Maldague B, Esselinckx W, Noel H, De Nayer P, Vincent A (1989). Spontaneous healing of aneurysmal bone cysts. A report of three cases. J Bone Joint Surg Br.

[9] Boriani S, De Iure F, Campanacci L, Gasbarrini A, Bandiera S, Biagini R, Bertoni F, Picci P (2001). Aneurysmal bone cyst of the mobile spine: report on 41 cases. Spine (Phila Pa 1976).

[10] Saccomanni B (2008). Aneurysmal bone cyst of spine: a review of literature. Arch Orthop Trauma Surg.

